# Case Report: A concomitant case of eosinophilic granuloma of the spine and *Fasciola hepatica* infection of the liver in a young patient, and review of the literature

**DOI:** 10.3389/fsurg.2025.1720707

**Published:** 2026-02-03

**Authors:** Jinri Zhang, Haoxian Li, Yingcong Wu, Dinghao Zhang, Chuhai Xie, Long Ling, Hailan Hu, Qi Liu

**Affiliations:** 1 Department of Orthopaedic Surgery, The Second Affiliated Hospital of Guangzhou Medical University Guangzhou, Guangdong, China; 2 Pain Department, Dongguan Binhaiwan Central Hospital Dongguan, Guangdong, China

**Keywords:** eosinophilic granuloma, *Fasciola hepatica*, literature review, spine, liver

## Abstract

Eosinophilic granuloma (EG) is a benign osteolytic bone lesion and is a localized form of Langerhans cell histiocytosis, most commonly involving the skeletal system. EG is a rare tumor, and involvement of the spine is low. Fascioliasis is a zoonotic disease caused by Fasciola species. The concomitant occurrence of EG and *Fasciola hepatica* infection is very rare. We report the clinical case of a young man (22 years old) who presented with acute pain in the dorsum. Magnetic resonance imaging revealed a T9 vertebra lesion that was hypointense on T1-weighted images and hyperintense on T2-weighted images. Computerized tomography demonstrated an osteolytic bone lesion in the T9 vertebra. Meanwhile, Doppler ultrasound of the liver displayed multiple echo groups in the bile duct, which suggested *Fasciola hepatica* infection. The lesion was removed by posterior surgery, during which gray-brown granulation tissue was noted in the T9 vertebra. Histological and immunohistochemical indices confirmed the diagnosis of the EG. Ectopic spinal localization of Fasciola was reported. This case presented a concomitant EG of the T9 vertebra and *Fasciola hepatica* infection in the liver, which must be distinguished by surgical resection and pathological evaluation.

## Introduction

Eosinophilic granuloma (EG) is a benign osteolytic bone lesion, derived from mononuclear and dendritic precursor cells. These cells are primarily located in the bone marrow but have the ability to migrate into tissues, where they act as antigen-presenting cells to T lymphocytes. The proliferation of Langerhans cells may be triggered by viral infections, bacterial infections or immune dysfunction, leading to increased levels of cytokines like interleukin-1 and interleukin-10 (1), initially described by Otani and Ehrlich (2) and Lichtenstein and Jeffe (3) as a destructive lesion with large numbers of eosinophilic cells.

EG is a rare disease affecting the spine, constituting <1% of spinal column tumors (4, 5). It is most commonly seen in the thoracolumbar spine and is often considered a disease of childhood, seen mostly in the first decade of life (6–8). In 79% of eosinophilic granuloma cases, the disease presents as a solitary lesion, while approximately 7% exhibit multifocal manifestations and 14% are associated with other forms of the disease (9).

Fascioliasis is an important emerging food-borne disease caused by the trematode species *Fasciola hepatica* and *F. gigantica* (10). Humans and animals typically become infected by ingestion of metacercariae present in wild or cultured freshwater vegetables, although infection by ingestion of contaminated water is also possible (11). After ingestion, the immature flukes exit the gastrointestinal tract, through the wall of the stomach or duodenum, and migrate through the peritoneal cavity to the liver (12).

However, the parasites can also migrate to the spinal canal and vertebrae. Vatsal et al. reported a case of ectopic infestation of the spine by *Fasciola hepatica* (13). In the present article, we report a case of concomitant eosinophilic granuloma.

## Case presentation

**Figure 1:**
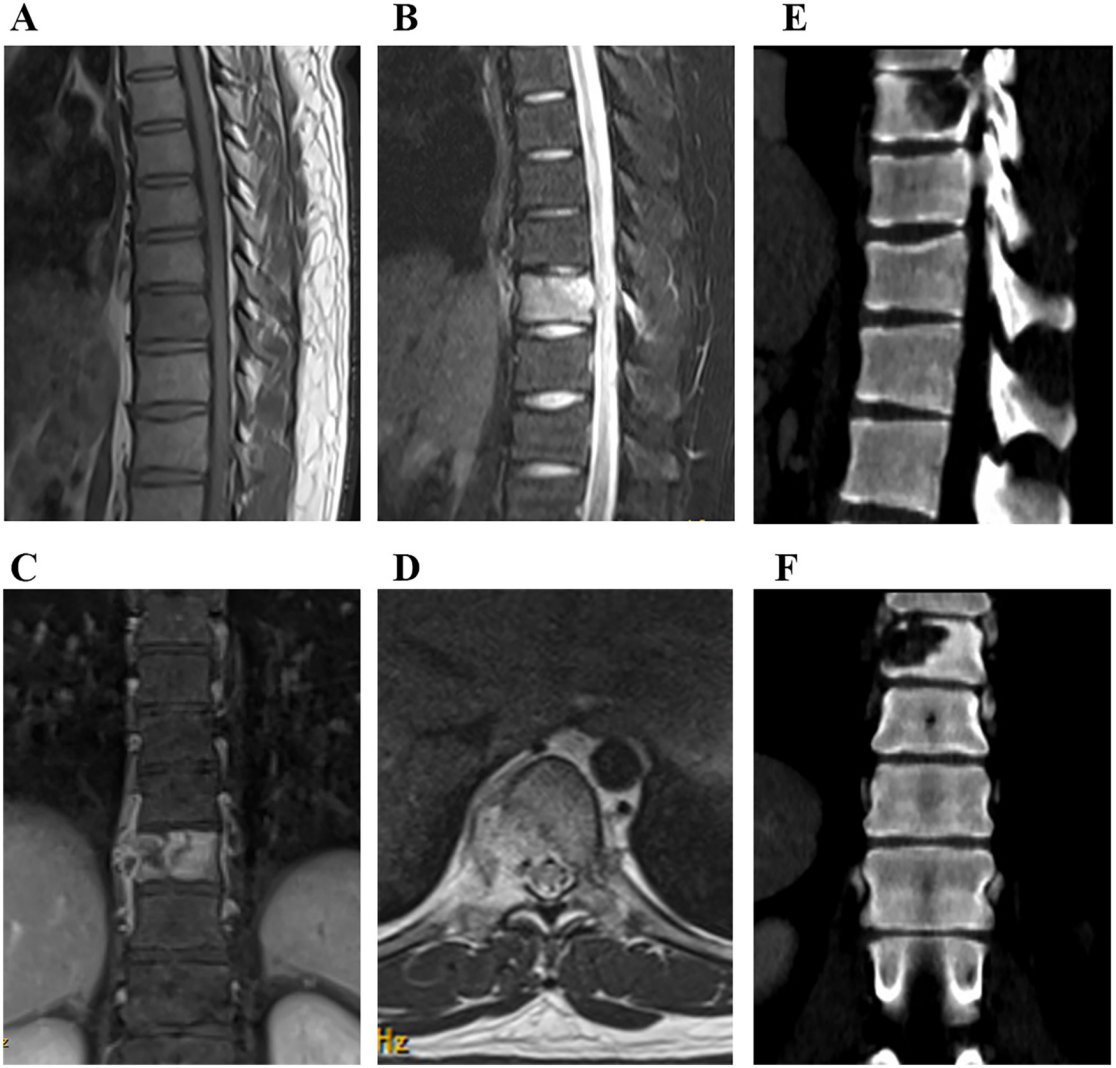
Preoperative MRI **(A–D)** and CT **(E,F)** examinations. An occupation lesion, hypointense on T1-weighted images and hyperintense on T2-weighted images, was detected in the ninth thoracic vertebrae.

**Figure 2:**
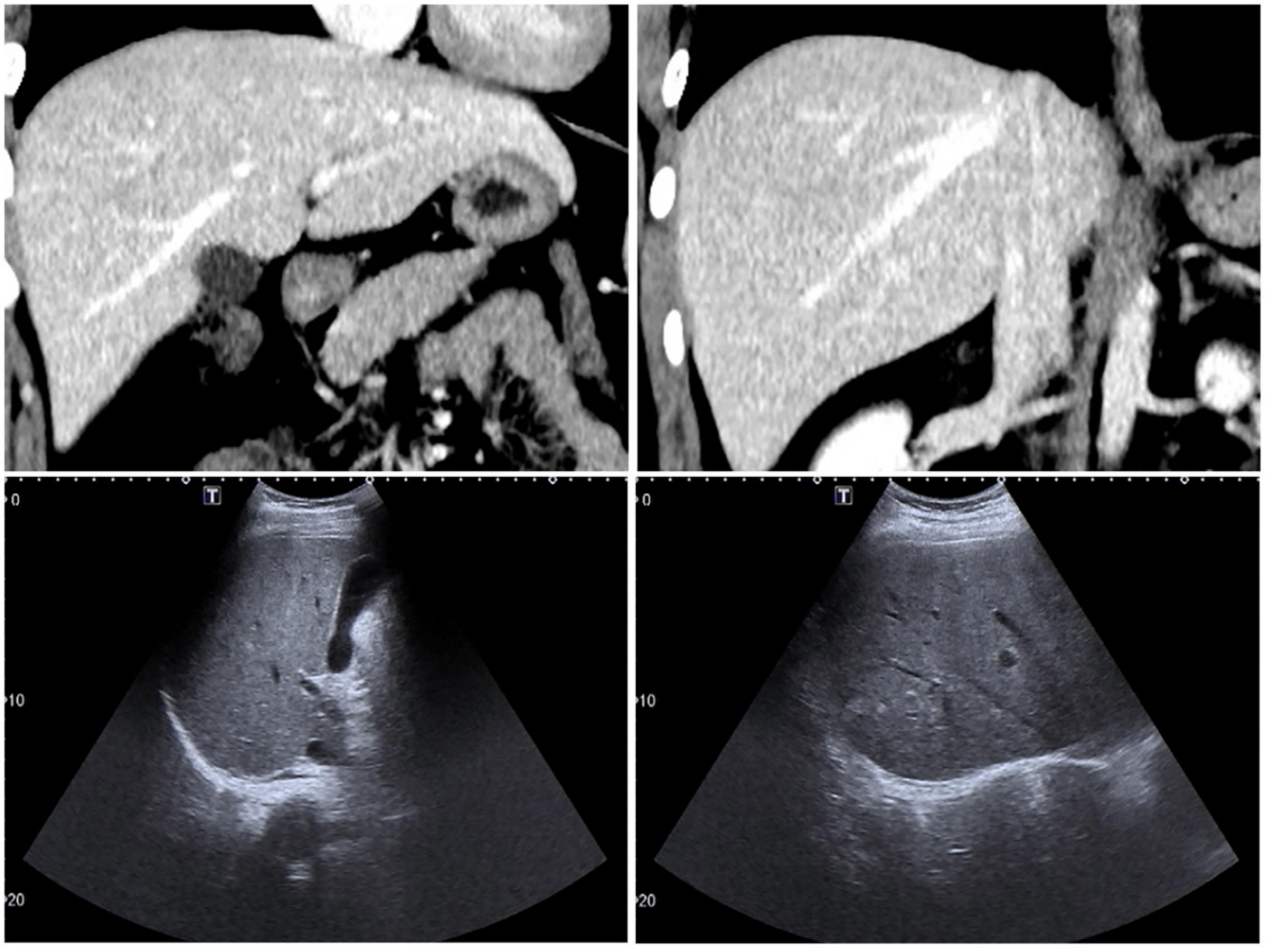
Ultrasound examination of liver.

In January 2019, a 22-year-old man presented with complaints of pain in the thoracic spine without lower extremity weakness or numbness. The pain worsened with spinal activity. Past history of cough, respiratory symptoms, family history of cancer, or evidence of any other illness was negative. However, the patient had a history of eating raw fish. An MRI scan performed at another hospital revealed an occupying lesion hypointense on T1-weighted images and hyperintense on T2-weighted images in the ninth thoracic vertebrae (Figure 1). The hematological index showed significantly elevated eosinophils, erythrocyte sedimentation rate, and C-reactive protein (Table 1). Liver function index was also abnormal, with elevated alkaline phosphatase, alanine aminotransferase, and *γ*-glutamyltransferase (Table 2). No parasite eggs were detected in the stool sample, and the tumor index showed no abnormalities (Table 3). In addition, liver ultrasonography suggested *Fasciola hepatica* infection (Figure 2). Based on these findings, a therapeutic and diagnostic lesion clearance was planned.

**Table 1:** Routine blood test before surgery.

Blood routine	Results	Range
Red blood cell count (1,012/L)	5.4	4.3–5.8
White blood cell count (109/L)	9.2	3.5–9.5
Hemoglobin (g/L)	144	130–175
Platelet count (109/L)	316	125–350
Neutrophil absolute value (109/L)	5.65	1.8–6.3
Lymphocyte absolute value (109/L)	2.13	1.1–3.2
Eosinophil absolute value (109/L)	0.66	0.02–0.52**#**
Erythrocyte sedimentation rate (mm/h)	43	0–15**#**
C reactive protein (mg/L)	29.3	0–8.3**#**

**Table 2:** Liver function test before surgery.

Liver function	Results	Range
Alanine aminotransferase (U/L)	93	9–50#
Aspartate aminotransferase (U/L)	22	15–40
Alkaline phosphatase (U/L)	165	40–125#
*γ*-Glutamyltransferase (U/L)	507	10–60#
Total protein (g/L)	77	65–85
Albumin (g/L)	39.1	40–55$
Total bilirubin (µmol/L)	18.8	6.0–22.0
Direct bilirubin (µmol/L)	9.6	0.0–6.0#
Indirect bilirubin (µmol/L)	9.2	6.0–22.0

**Table 3:** Tumor index test before surgery.

Tumor index	Results	Range
Alpha-fetoprotein (µg/L)	1.61	0.89–8.78
Tumor carbohydrate antigen-19.9 (U/mL)	<2.00	0–37
Carcinoembryonic antigen (µg/L)	1.55	0–5

## Intervention

**Figure 3:**
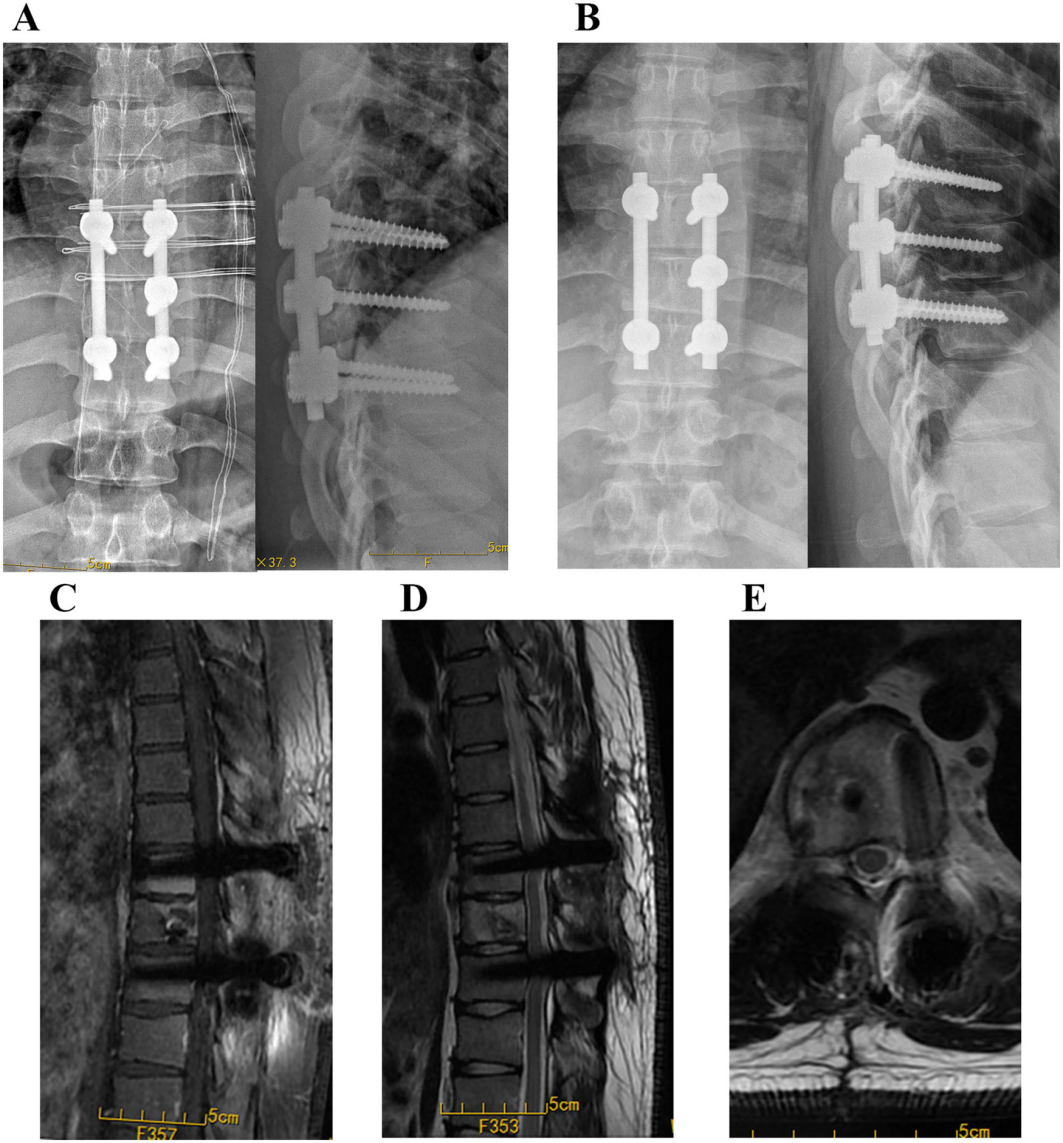
Postoperative follow-up imaging examination. **(A)** The X-ray of the spine at the 1 month postoperation. **(B–E)** The X-ray and MRI images at the 6 months postoperation.

The patient underwent T9 laminectomy and T8-11 fixation (Figure 3A). During removal of the gray-brown granulation tissue, autogenous iliac bone was grafted into the lesion, and all the excised tissues were sent for histopathological analysis. Postoperatively, the patient’s dorsum pain was relieved. At the 6-month follow-up, X-ray of thoracic spine demonstrated a good outcome with a stable fixation and good fusion (Figures 3B–E).

## Histopathological examination

**Figure 4:**
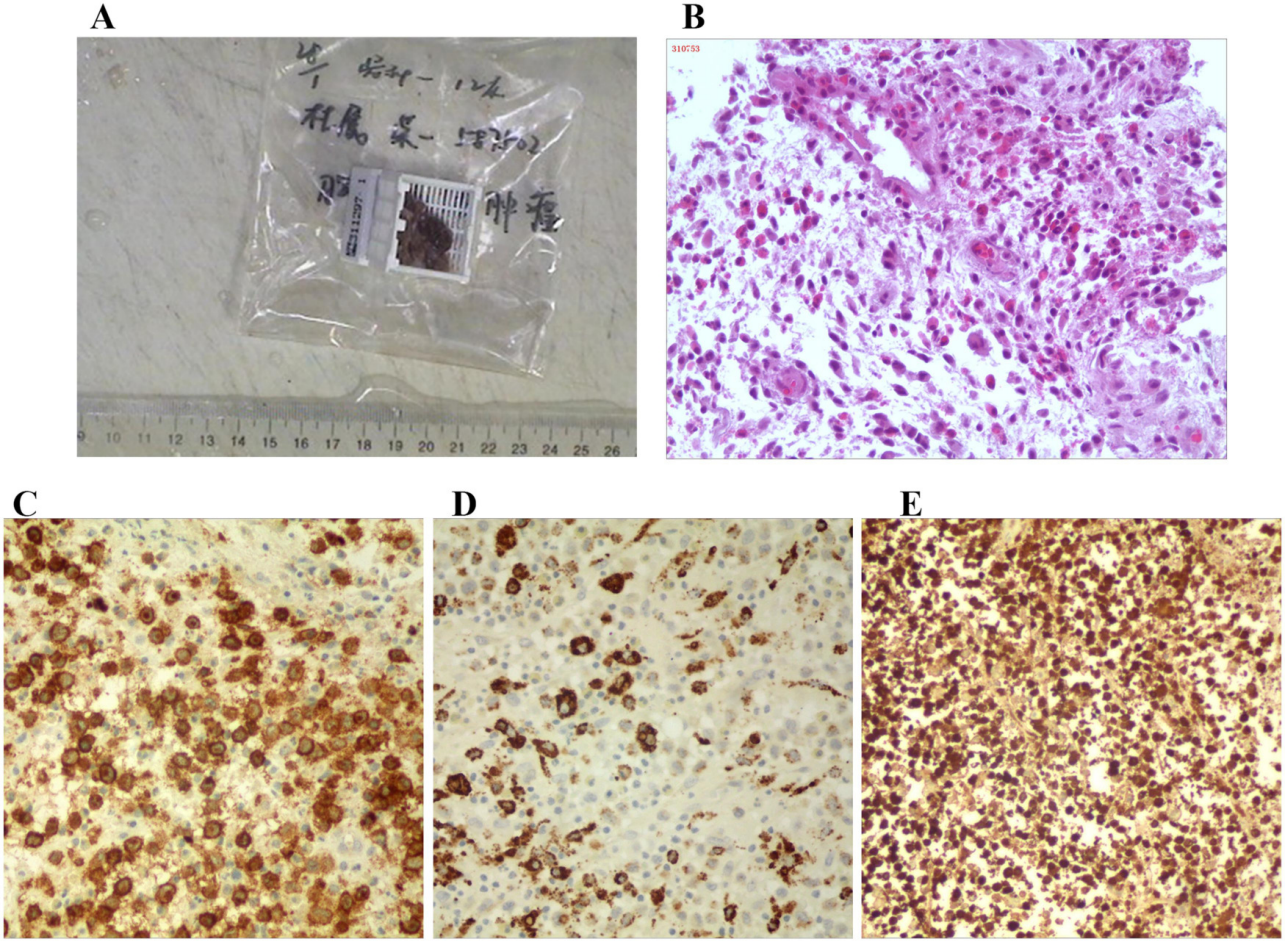
The pathological results. **(A)** The general view of the lesion. **(B)** The hematoxylin–eosin staining of lesion. **(C–E)** The CD1a, CD68, and S-100 staining of the lesions.

Tissue biopsy is necessary for histological diagnosis when clinical and radiological manifestations are ambiguous (14–16). Grossly, these lesions appeared off-white to reddish-brown with intervening bony spicules (Figure 4A). Hematoxylin–eosin staining highlighted proliferation of eosinophils, histiocytes, lymphocytes, and macrophages, as well as aggregates of dendritic Langerhans cells (Figure 4B). We further clarified the diagnosis through immunohistochemistry with positive reactions for CD1a, CD68, and S-100 (Figures 4C–E).

## Discussion and literature review

This article presents a concomitant case of eosinophilic granuloma of the spine and *Fasciola hepatica* infection of the liver in a young patient. Eosinophilic granuloma in bone is a histiocytosis confined to bone tissue, which is a type of Langerhans cytosis characterized by bone destruction, histiocytosis, and eosinophil infiltration.

EG is a rare disorder with an incidence of four to five cases per million per year in children under15 years and an incidence of one to two cases per million per year in adults (1, 17). Children aged between 5 and 15 years are most frequently affected, making EG predominantly a disease of childhood and adolescence, and rare in adults (18, 19). EG incidence in the spine can present as single or multiple lesions, with single lesions being more common. The highest incidence of EG is observed in the thoracic spine at 54%, followed by the lumbar spine at 35% and cervical spine at 11% (20). Spinal EG lacks typical clinical manifestations and mainly presents as local pain. In cases of severe disease progression, pathological fractures and spinal deformities occur, and neurological symptoms appear when the spinal cord is compressed.

X-ray imaging is the first choice for spinal EG examination, as it provides important reference value for establishing diagnosis. Early X-ray reveals bone destruction in the central area of the vertebral body, which manifests as focal or cystic osteolytic bone lesion, eventually forming a “flat vertebra” or coin-like vertebra (18). The “flat vertebra” is characteristic of EG in the spine, which is more common in young patients. Prasad et al. reported that only 40% of adult spinal EG cases exhibited this typical manifestation (21). CT and MRI provide superior detail of spinal EG lesions, clearly exhibiting the scope and characteristics of bone destruction and the relationship between soft tissue masses, lesions, and adjacent structures (22).

Spinal parasites are relatively rare and may present with typical symptoms such as back pain, numbness, weakness, or bowel/bladder incontinence. Infections such as cysticercosis, schistosomiasis, toxoplasmosis, and echinococcus can involve the spine (23–26). These cases typically involve the spinal canal, presenting signs of spinal cord compression. Viljoen et al. (27) reported a case where the patient complained of severe back pain radiating down his right leg. Radiographs demonstrated complete destruction of the L1 vertebral body, with extension across the intervertebral disc into the T12 vertebral body, as well as anterior subligamentous spread. This was subsequently diagnosed as hydatid disease. A 40-year-old woman presented with sensory loss and progressive lower-limb weakness. Imaging revealed a lytic lesion at the T5 thoracic vertebral level, and pathological examination confirmed the diagnosis of thoracic vertebral body cysticercosis (28).

*Fasciola hepatica* infection in humans is a secondary zoonotic disease that typically affects the hepatobiliary and pulmonary systems, causing significant morbidity and mortality. The spine is a rare site for ectopic localization of *Fasciola hepatica*. Devendra et al. reported a case of ectopic fascioliasis in the dorsal spine (13). The patient presented with gradual-onset paraplegia with bladder and bowel involvement. MRI revealed an epidural mass lesion that was isointense on T1-weighted images and hyperintense on T2-weighted images, extending from the T4 to the T7 vertebrae with extradural cord compression. Morphological and histological analyses confirmed the parasite to be *Fasciola hepatica*.

Spinal eosinophilic granuloma and parasitic infections are relatively rare, and exhibit similar symptoms. They often manifest as lower back pain, leg pain, numbness, weakness, or bowel/bladder incontinence. In the present case, the patient exhibited back pain at the T9 level with no symptoms of spinal cord compression. Radiological examination revealed vertebral body/spinal canal lesions, or vertebral fracture. Pathological examination, which is the golden standard for diagnosis, confirmed T9 eosinophilic granuloma through surgical excision and histological staining.

The pathogenesis of EG remains uncertain and several hypotheses have been proposed regarding its etiology. EG is considered a disorder of the immune system, representing a hypersensitivity reaction with stimulation of the histiocyte–macrophage system. The immaturity of the immune system is thought to directly influence the severity of the disease (29, 30). Kaplan et al. indicated that eosinophilic granuloma in the liver may be closely related to visceral larva migrans of parasites (31). Studies have demonstrated that *Fasciola hepatica* and its excretory/secretory antigens have stimulated eosinophil maturation in the bone marrow of mice (32). In the present case, it remains unclear whether the EG of the T9 vertebra was caused by *Fasciola hepatica* infection.

## Conclusion

This report describes a concomitant case of EG of the spine and *Fasciola hepatica* infection of the liver in a young man. Both EG and parasitic infections can involve the spine and present with similar symptoms. Surgical intervention and pathological examination remain the gold standard for definitive diagnosis. Notably, the correlation between eosinophilic granulomas and liver trematodes remains uncertain and is still under discussion.

## Data Availability

The original contributions presented in the study are included in the article/Supplementary Material; further inquiries can be directed to the corresponding authors.
